# QUANTIFYING THE BURDEN OF HOSPITALIZED DAYS IN MEDICARE BENEFICIARIES WITH MULTIMORBIDITY

**DOI:** 10.1093/geroni/igz038.3365

**Published:** 2019-11-08

**Authors:** Melissa Y Wei, Nicholas Tilton, Kenneth J Mukamal

**Affiliations:** 1 Division of General Medicine, University of Michigan, Ann Arbor, Michigan, United States; 2 Division of General Medicine, Beth Israel Deaconess Medical Center and Harvard Medical School, Brookline, Massachusetts, United States

## Abstract

Multimorbidity predicts several health outcomes including physical and cognitive functioning and mortality. Multimorbidity also predicts healthcare burden, but this has not been studied using a patient-centered measure that weights conditions by their impact on physical functioning. Health and Retirement Study participants were continuously enrolled in Medicare Parts A/B 1-year before and after the 2012-2013 HRS interview. Medicare claims were used to compute ICD-coded multimorbidity-weighted index (MWI-ICD) by summing physical functioning-weighted conditions. Given excess observations of zero hospital days (78.1%), we used zero-inflated Poisson regression to examine the association between multimorbidity and hospitalized days. First, logit models predicted membership into the zero-coded “no hospitalizations” group. Second, Poisson models predicted hospital days for participants not in the zero-coded group. We converted adjusted regression coefficients to odds ratios to report odds of zero hospitalized days. To compare model fit between MWI-ICD and simple disease count we used AICs. The final sample N=5201 participants had mean age 77.6+/-11.6 years, MWI-ICD 16.5+/-11.6, and 1.9+/-6.0 (range 0-90) hospitalized days. Each 1-point increase in MWI-ICD was associated with 4.3% decreased odds of zero hospitalized days (OR=0.96, 95%CI: 0.95-0.96) and 2% increased number of expected hospitalized days (IRR=1.02, 95%CI: 1.01-1.03) over one year in adjusted models. MWI-ICD had a lower AIC than simple disease count. Multimorbidity measured with MWI-ICD was associated with a decreased odds of zero hospitalized days and an increased number of expected hospitalized days. Multimorbidity contributes greatly to patient burden through increased hospitalization and is better captured through an index weighting conditions to physical functioning.

